# Muscle sympathetic nerve activity in frontotemporal lobar degeneration is similar to amyotrophic lateral sclerosis

**DOI:** 10.1007/s10286-015-0321-y

**Published:** 2015-11-25

**Authors:** Kazumasa Shindo, Michiaki Miwa, Fumikazu Kobayashi, Takamura Nagasaka, Yoshihisa Takiyama

**Affiliations:** Department of Neurology, University of Yamanashi Hospital, 1110 Tamaho, Yamanashi, 409-3898 Japan

**Keywords:** Amyotrophic lateral sclerosis, Frontotemporal lobar degeneration, Muscle sympathetic nerve activity, Blood pressure, Sympathetic overactivity

## Abstract

**Purpose:**

To determine whether frontotemporal lobar degeneration (FTLD) is associated with similar cardiovascular autonomic dysfunction to that seen in amyotrophic lateral sclerosis (ALS), we compared cardiovascular parameters between ALS patients and patients with FTLD.

**Methods:**

In ten patients with FTLD (mean age ± SD: 71.6 ± 4.6 years) and 12 patients with ALS (mean age ± SD: 71.4 ± 4.6 years), MSNA (using microneurography), heart rate (HR), and blood pressure (BP) were recorded simultaneously.

**Results:**

MSNA was significantly higher in both groups of patients compared with the controls (*p* < 0.01), while there were no significant differences in MSNA between the patients with FTLD and those with ALS. During head-up tilt, changes in HR, BP, and the frequency of MSNA bursts were smaller in the patients than in controls (*p* < 0.05 or *p* < 0.01).

**Conclusions:**

Patients with FTLD and ALS showed similar dysfunction of HR, BP, and sympathetic outflow to muscles.

## Introduction

Although traditionally, frontotemporal lobar degeneration (FTLD) and amyotrophic lateral sclerosis (ALS) have been considered distinct disorders (the first one featuring cognitive dysfunction and language disturbance and the latter featuring progressive muscle atrophy and weakness), growing evidences suggest that FTLD signs can be seen in patients primarily diagnosed with ALS, implying clinical overlap among these two disorders [3, 11]. Similarly, pathological and genetic studies suggest that both disorders are intimately linked [1–3, 5, 10]. Investigations of cardiovascular autonomic function in ALS have disclosed increased resting heart rate (HR), increased daily fluctuations of blood pressure (BP), and increased sympathetic outflow to muscles and skin [6, 9, 12, 13, 16]. Preliminary studies of cardiovascular autonomic function in patients with FTLD show some similarities to those in ALS patients, including increased daily fluctuation of BP and orthostatic hypotension in some patients [8, 10, 14, 15]. Accordingly, we aimed to determine whether MSNA is abnormal in ALS and FTLD, and whether abnormalities of cardiovascular autonomic function are similar in the two groups.

## Materials and methods

### Subjects

A cross-sectional study was performed in patients with FTLD and ALS regularly going to our hospital. The FLTD group was ten patients with FTLD (3 men and 7 women; mean age ± SD: 71.6 ± 4.6 years; range: 65–79 years). The mini mental state examination (MMSE) score ranged from 16 to 22 (mean: 19.2 ± 2.3), and all patients were diagnosed as having progressive nonfluent aphasia (PA) according to the consensus criteria of Neary et al. [4]. The interval from the onset of PA to this study ranged from 1 to 3 years. None of them had symptoms related to the cardiovascular system.

The ALS group was 12 patients with ALS (4 men and 8 women; mean age ± SD: 71.4 ± 4.6 years; range: 64 to 78 years), in whom Jablecki disability score ranged from 8 to 29 (mean: 16.9 ± 6.4). All 12 patients had clinically definite sporadic ALS according to the El Escorial criteria. The initial symptoms were cervical in 9 patients and bulbar in 3 patients. None of them had pain or neuropathy. The MMSE score ranged from 26 to 30 (mean: 28.3 ± 1.5) and cognitive impairment was not observed in any of the patients based on the results of the Wechsler Memory Scale-Revised, Wisconsin Card Sorting Test, and Boston Naming Test. Nerve conduction studies were normal, while electromyography revealed neurogenic changes of the bulbar muscles and the muscles in all extremities. All patients had mild-to-moderate bulbar signs and were able to walk unaided. PaO_2_ was greater than 70 Torr, PaCO_2_ was less than 45 Torr, and forced vital capacity exceeded 70 % of the predicted value in all patients. The interval from the onset of ALS to this study ranged from 8 to 42 months. None of the patients had symptoms related to the cardiovascular system.

None of the patients had dyspnea at rest, and none of them had concurrent disorders such as hypertension, cardiovascular disease, or cerebrovascular disease. Drugs that might have an influence on the autonomic nervous system, such as muscle relaxants, vasodilators, narcotics, or antidepressants, were discontinued 2 days before the study.

The control group consisted of 16 healthy volunteers (5 men and 11 women; mean age ± SD: 71.9 ± 5.9 years; range: 62–79 years). Their MMSE scores ranged from 27 to 30 (mean: 28.6 ± 1.2).

### MSNA, HR, and BP recording

Muscle sympathetic outflow was recorded by microneurography after written informed consent was obtained from each subject. The procedure was approved by the local IRB. Subjects were tested in the supine position. MSNA was recorded directly from the peroneal nerve in the right popliteal fossa using tungsten microelectrodes. Neurograms were obtained by using the methods and instruments described previously [13]. Identification of MSNA was based on the following three criteria: (1) spontaneous and pulse-synchronous rhythmic burst discharges; (2) modulation of the bursts by spontaneous respiration; and (3) marked accentuation of the bursts by an increase of intrathoracic pressure such as with the Valsalva maneuver. The electrodes were connected to a preamplifier (DAM50, WPI, Sarasota, FL) using a gain of 100 and to an amplifier (AVM-10, Nihon Kohden, Japan) with a gain of 500. A band-pass filter of 500–2000 Hz was used. To obtain the mean voltage neurogram, the filtered neurogram was fed into an RC integrating unit (EI-601G, Nihon Kohden, Japan) using a time constant of 0.1 s.

The electrocardiogram (ECG) was recorded using chest wall electrodes and HR was monitored from the ECG. BP was continuously recorded and measured with a tonometric device (Jentow, Nihon Kohlin, Japan). The tonometric sensor was attached to the wrist, which was kept at the level of the right atrium. The ECG, BP, and MSNA were monitored with an oscilloscope (VC-10, Nihon Kohden, Japan) and data were recorded simultaneously on a thermal array recorder (RTA-1200, Nihon Kohden, Japan) at a paper speed of 5 mm/s. After a 15-min acclimatization period, recording of resting data was performed for 30 min. In order to compare the response of MSNA to baroreflex modulation, the patients and control subjects were positioned with the head tilted up on an electric tilt table set at an angle of 30° when this was possible. At this angle, ALS patients with muscular weakness in the lower extremities could still support themselves unaided. The tilt maneuver was performed in all subjects, except for 2 ALS patients whose microelectrodes fell out immediately after tilting. HR, BP, and MSNA were monitored simultaneously for 30 min at rest and during 30° head-up tilt.

### Measurement and analysis

MSNA was quantified by calculating the frequency of bursts of sympathetic activity (MSNA burst frequency: bursts/100 heart beats). Data on the HR, BP, and MSNA were averaged over 1-min periods for a total of 10 min. Investigators who were blinded to the groups of the subjects identified MSNA bursts by inspection of the mean voltage neurogram. For assessment of BP, mean BP (MBP) was calculated as diastolic BP+1/3 × (systolic BP − diastolic BP). Changes of HR, BP, and MSNA were expressed as delta values (Δ).

The data were entered in a computer (PD-E 550, Sharp, Japan) for analysis. Results are expressed as the mean ± SD. Differences were evaluated by the Kruskal–Wallis test and post hoc comparisons with separate Mann–Whitney tests (SPSS 19. 0.1 for Mac, IBM Inc.). *p* < 0.05 was considered to indicate statistical significance.

## Results

**Fig. 1:**
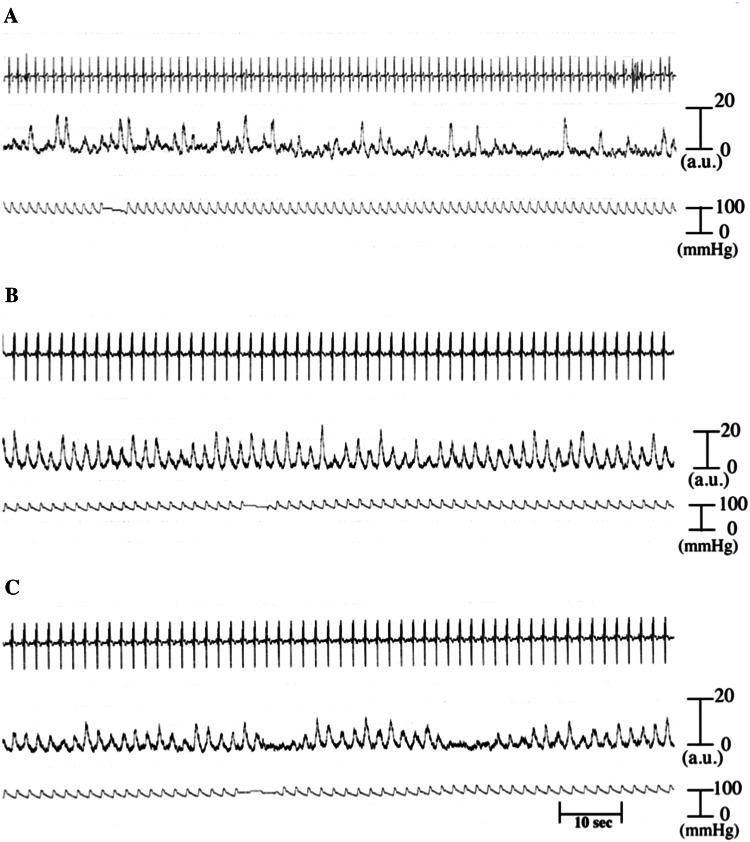
Representative recordings in a 79-year-old healthy male volunteer (**a**), a 69-year-old male with FTLD (**b**) and a 69-year-old female with ALS (**c**) at rest (*top trace* electrocardiogram, *middle trace* integrated muscle sympathetic nerve activity, *bottom trace* blood pressure). *a*.*u*. arbitrary units

Representative recordings of MSNA obtained in patients with FTLD and ALS, as well as in a healthy control subject, are displayed in Fig. 1. Both patients showed an increase of MSNA bursts compared with the control subject. The mean values of HR, MBP, and MSNA averaged over 1-min periods for a total of 10 min in the three groups are listed in Table 1. There were no differences in resting HR and BP between the patients and controls (Table 1). While the MSNA burst frequency was significantly higher in both groups of patients compared with the controls (*p* < 0.01) (Table 1), there were no significant differences in MSNA between the patients with FTLD and those with ALS. During head-up tilt, changes in HR, BP, and the MSNA burst frequency were smaller in the patients than in controls (*p* < 0.05 or *p* < 0.01) (Table 1).

**Table 1:** Comparison of mean values between patients with FTLD, ALS and control subjects in each parameter

	FTLD (*n* = 10)	ALS (*n* = 12)	Controls (*n* = 16)	*p* values
Age (years)	71.6 ± 4.6	71.4 ± 4.6	71.9 ± 5.9	0.8639
At rest				
HR (/min)	73.1 ± 12.2	73.5 ± 11.3	76.5 ± 10.6	0.4820
MBP (mmHg)	92.6 ± 11.6	94.9 ± 19.3	93.0 ± 24.2	0.8898
MSNA (b/100 HB)	83.4 ± 13.9^a^	81.2 ± 12.8^b^	62.8 ± 14.1	0.0013
Head-up tilting				
ΔHR (/min)	+2.5 ± 1.2^c^	+3.4 ± 2.2^d^	+7.9 ± 4.2	0.0376
ΔMBP (mmHg)	+11.5 ± 3.2^c^	+9.0 ± 1.0^d^	+15.4 ± 10.8	0.0389
ΔMSNA (b/100 HB)	+2.8 ± 1.0^a^	+3.8 ± 2.1^b^	+7.9 ± 4.2	0.0057

*HR* heart rate, *MBP* mean blood pressure, *MSNA* muscle sympathetic nerve activity, *b* bursts, *HB* heart beats, Δ increment for each parameter, *FTLD* frontotemporal lobar degeneration, *ALS* amyotrophic lateral sclerosis, *p* values *p* values for Kruskal–Wallis test

^a^ *p* < 0.01 (FTLD vs. controls)

^b^ *p* < 0.01 (ALS vs. controls)

^c^ *p* < 0.05 (FTLD vs. controls)

^d^ *p* < 0.05 (ALS vs. controls)

## Discussion

The present study showed that resting MSNA was increased in patients with FTLD or ALS compared with age-matched controls, although there were no significant differences in HR and BP. The two groups of patients showed no significant differences in HR, BP, or MSNA either at rest or during head-up tilt. The reason why elevated MSNA was not associated with an increase of BP or HR is possibly that a continuous increase of sympathetic outflow to muscles causes down-regulation of the vasoconstrictor response of intramuscular vessels because of reduced sensitivity of peripheral α-adrenoceptors, as suggested by the blunted response of BP to intravenous administration of norepinephrine reported previously in ALS [12, 13, 16].

Several researchers have investigated autonomic function related to the cardiovascular system and muscle sympathetic outflow in ALS by performing physiological and pharmacological studies. The findings included an increase of resting HR, a reduced BP response to standing, increased daily fluctuation of HR or BP, and chronotropic insufficiency and lack of norepinephrine release upon head-up tilt [12, 16]. However, most investigators have found little or no impairment of cardiovascular autonomic function in relation to regulation of HR and BP. Regarding sympathetic regulation of BP, two studies indicated that resting MSNA is significantly increased in ALS patients compared with healthy subjects and patients with other neuromuscular diseases [6, 13], while the another study showed that the baseline blood pressure and MSNA variables did not differ between ALS patients and healthy controls [7]. In addition, sympathetic overactivity was confirmed by cardiac MIBG scintigraphy [17]. It has been proposed that sympathetic overactivity due to the central mechanism contributes to autonomic dysfunction in ALS [6, 12, 13, 16].

Some investigators have mentioned autonomic symptoms in patients with FTLD, including urinary incontinence, hypotension, and increased daily fluctuation of BP [8, 10, 14, 15]. Passant et al. and Struhal et al. reported that patients with frontotemporal dementia (a subtype of FTLD) exhibited a significantly increased frequency of orthostatic hypotension [8, 15]. Although there is a paucity of other reports on cardiovascular function in FTLD, and secondary causes such as dehydration, anemia, and deconditioning may contribute to the occurrence of orthostatic hypotension, the present study revealed that changes of HR, BP, and MSNA during head-up tilt were significantly attenuated in FTLD. It is possible that sympathetic overactivity detected in the present study contributes to HR elevation, orthostatic hypotension, and increased daily fluctuation of BP in patients with ALS and FTLD. Furthermore, symptoms associated with HR or BP might be masked by dementia or aphasia in patients with FTLD. Although the central mechanism involved remains unknown, pathological changes of the limbic system in the medial temporal lobe (frequently affected in FTLD) might have the effect on the cardiovascular system. In conclusion, further investigations of cardiovascular function in patients with other subtypes of FTLD will be needed to clarify whether or not there are similarities of autonomic dysfunction between ALS and FTLD.

## Abbreviations

ALS: Amyotrophic lateral sclerosis
FTLD: Frontotemporal lobar degeneration
PA: Progressive nonfluent aphasia
HR: Heart rate
MBP: Mean blood pressure
MSNA: Muscle sympathetic nerve activity
IRB: Institutional review board
